# Rate and Nearly-Lossless State over the Gilbert–Elliott Channel

**DOI:** 10.3390/e27050494

**Published:** 2025-05-02

**Authors:** Amos Lapidoth, Ligong Wang

**Affiliations:** Department of Information Technology and Electrical Engineering, ETH Zurich, 8092 Zurich, Switzerland

**Keywords:** causal, Gilbert–Elliott channel, noncausal, rate-and-state capacity, state information, strictly causal

## Abstract

The capacity of the Gilbert–Elliott channel is calculated for a setting in which the state sequence is revealed to the encoder and is, along with the transmitted message, to be conveyed to the receiver with a vanishing symbol error rate. Said capacity does not depend on whether the state sequence is provided to the encoder strictly causally, causally, or noncausally. It can be achieved using a Block-Markov coding scheme with backward decoding.

## 1. Introduction

The Gilbert–Elliott channel [1,2,3,4] is a binary-input, binary-output finite-state channel [5,6,7,8], which was proposed as a simple model for digital communications with bursty errors. Its state sequence is a stationary two-state Markov process whose Time-*i* state $S_{i}$ takes values in the set S={0,1}. When the Time-*i* state $S_{i}$ is 0, the Time-*i* channel output $Y_{i}$ is the result of feeding the Time-*i* input $x_{i}$ through a Binary Symmetric Channel (BSC) of crossover probability $\epsilon_{0}$; when $S_{i}$ is 1, the output $Y_{i}$ is the result of feeding $x_{i}$ through a BSC of crossover probability $\epsilon_{1}$. Alternatively, we can describe the output sequence as being the componentwise mod-2 addition of the input sequence with a noise sequence $\left\{Z_{i}\right\}$, where the latter is a binary two-state Hidden Markov process.

The capacity of the Gilbert–Elliott channel is achieved by having the input sequence comprise Independent and Identically Distributed (IID) random bits. It is given, in bits, by $1-H(\left\{Z_{i}\right\})$, where $H(\left\{Z_{i}\right\})$ denotes the entropy rate of the noise $\left\{Z_{i}\right\}$ [3]. If the state sequence is revealed to the decoder, then capacity is still achieved by the above input distribution, but it now equals the weighted average of the capacities of the BSCs, namely, $\pi_{0}\;\left(1-H_{b}\left(\epsilon_{0}\right)\right)+\pi_{1}\left(1-H_{b}\left(\epsilon_{1}\right)\right)$. Here, $\pi_{1}=1-\pi_{0}$ denotes the probability that $S_{i}$ is 1, and $H_{b}\left(\cdot \right)$ is the binary entropy function. Throughout this paper, logarithms are to base 2, and information is measured in bits.

Here, we study the case where the state sequence is revealed not to the decoder but to the encoder. We do not, however, seek the Shannon capacity but rather the “rate-and-nearly-lossless-state” capacity, where the decoder wishes to recover not only the transmitted message but also the state sequence. We thus require that the state sequence be conveyed to the decoder with a vanishing symbol error rate. This requirement is weaker than the requirement that the probability of the receiver correctly recovering the entire state sequence tends to one. Neither do we consider a general (nonzero) distortion constraint, as studied for memoryless channels in [9,10].

We solve for this capacity and show that it does not depend on whether the state sequence is provided to the encoder strictly causally, causally, or noncausally. Moreover, it can be achieved using a Block-Markov coding scheme with backward decoding.

## 2. The Gilbert–Elliott Channel

The Gilbert–Elliott channel has binary input, output, and state alphabets: X=Y=S={0,1}. The evolution of the state is unaffected by the channel inputs: irrespective of the channel inputs, it forms a stationary time-homogeneous Markov chain of kernel

(1) $$\mathrm{Pr}\left[S_{i}=s_{i}|S^{i-1}=s^{i-1}\right]=\mathrm{Pr}\left[S_{i}=s_{i}|S_{i-1}=s_{i-1}\right]$$

and stationary distribution

(2) $$P_{S}=\left(\pi_{0},\pi_{1}\right)$$

by which we mean that

(3) $$P_{S}\left(1\right)=1-P_{S}\left(0\right)=\pi_{1}=1-\pi_{0}.$$

Above and throughout, we use $A^{i}$ to denote $A_{1},\ldots ,A_{i}$, and we use $A_{j}^{i}$ to denote $A_{j},\ldots ,A_{i}$. We denote the entropy rate of the state sequence $H(\left\{S_{i}\right\})$. It is given explicitly by [11]

(4) $$H\left(\left\{S_{i}\right\}\right)=H\left(S_{2}|S_{1}\right).$$

Given S=0, the channel from *X* to *Y* is a BSC with crossover probability $\epsilon_{0}$, whereas, given S=1, it is a BSC with crossover probability $\epsilon_{1}$. Here, $\epsilon_{0},\epsilon_{1}\in \left[0,1\right]$ are arbitrary known constants. Thus, if we define

(5) $$W\left(1-x|x,0\right)=1-W\left(x|x,0\right)=\epsilon_{0}$$

and

(6) $$W\left(1-x|x,1\right)=1-W\left(x|x,1\right)=\epsilon_{1},$$

then we can express the behavior of the channel given the state as

(7) $$\mathrm{Pr}\left[Y_{i}=y_{i}|X^{i}=x^{i},Y^{i-1}=y^{i-1},S^{i}=s^{i}\right]=W\left(y_{i}|x_{i},s_{i}\right).$$

## 3. The Rate-and-Nearly-Lossless-State Capacity

When discussing rate-*R* blocklength-*n* communications over the Gilbert–Elliott channel, we consider the message set $M=\{1,\ldots ,2^{nR}\}$ with $2^{nR}$ messages, one of which is to be conveyed to the receiver. The latter observes the output sequence $Y^{n}$ and attempts to recover the transmitted message *m* and the state sequence $S^{n}$. The decoder is thus specified by a function

(8) $$\phi :Y^{n}\to M\times S^{n},\;y^{n}\mapsto \left(\hat{m},{\hat{s}}^{n}\right).$$

As to the encoder, its structure depends on the manner in which the state information is revealed to it. In the strictly causal setting, the Time-*i* channel input may depend, not only on the transmitted message, but also on the past states; it is therefore denoted $X_{i}\left(m,S^{i-1}\right)$. The encoder is thus specified by *n* functions

(9) $$f_{i}:M\times S^{i-1}\to X,\;i=1,\ldots ,n,$$

with $X_{i}\left(m,S^{i-1}\right)$ being $f_{i}\left(m,S^{i-1}\right)$. In the causal case, the Time-*i* channel input is denoted $X_{i}\left(m,S^{i}\right)$, and the encoder is specified by *n* functions

(10) $$f_{i}:M\times S^{i}\to X,\;i=1,\ldots ,n,$$

with $X_{i}\left(m,S^{i}\right)$ being $f_{i}\left(m,S^{i}\right)$. Finally, in the noncausal case, the Time-*i* channel input is denoted $X_{i}\left(m,S^{n}\right)$, and the encoder is specified by one function

(11) $$f:M\times S^{n}\to X^{n},$$

with $X_{i}\left(m,S^{n}\right)$ being the *i*-th component of $f(m,S^{n})$.

We refer to an encoder/decoder pair as a “coding scheme”. The probability of error associated with a given coding scheme and a given message *m* is $\mathrm{Pr}[\hat{M}\neq m]$ calculated when the scheme’s encoder is used to transmit Message *m*, and the scheme’s decoder is used by the receiver. The average probability of error associated with a coding scheme is the arithmetic average of the probabilities of error associated with the different messages. It can be expressed as

(12) $$P_{e}≜\mathrm{Pr}\left[\hat{M}\neq M\right]$$

when the transmitted message *M* is drawn equiprobably from M.

The symbol error rate, or (expected) Hamming distortion, in reconstructing the state sequence is

(13) $$\beta ≜\frac{1}{n}\sum_{i=1}^{n}\mathrm{Pr}\left[{\hat{S}}_{i}\neq S_{i}\right]$$

again computed when the transmitted message *M* is drawn equiprobably from M.

In all cases, we say that a rate *R* is achievable if there exists a sequence of coding schemes indexed by the blocklength for which $P_{e}$ and β both tend to zero. The capacity is defined as the supremum of the achievable rates, with the understanding that, if no positive rate is achievable, then capacity is zero.

Our main result is the following theorem:

**Theorem** **1.** *Irrespective of whether the state information is provided to the encoder strictly causally, causally, or noncausally, if*

(14) $$1-\left(\pi_{0}H_{b}\left(\epsilon_{0}\right)+\pi_{1}H_{b}\left(\epsilon_{1}\right)\right)-H\left(\left\{S_{i}\right\}\right)$$

*is positive, then it equals the rate-and-nearly-lossless-state capacity of the Gilbert–Elliott channel; else, said capacity is zero.*

The proof is provided in the next section. Expression (14) can be interpreted as the result of subtracting the optimal average description length of the state sequence from the capacity of the channel when the receiver is cognizant of the state.

## 4. Proof of Theorem 1

### 4.1. Converse

We prove the converse part of the theorem under the assumption that the state sequence is provided to the encoder noncausally. Let *M* be uniform over M. We have

(15)H(Sn|M,Yn)≤H(Sn|S^n)(16)≤∑i=1nH(Si|S^i)(17)≤∑i=1nHbPr[Si^≠Si](18)≤nHb(β).

Here, (15) holds because ${\hat{S}}^{n}$ is a function of $Y^{n}$ and hence also of $\left(M,Y^{n}\right)$; (17) because ${\hat{S}}_{i}$ is binary and because conditioning reduces entropy; and (18) because $H_{b}\left(\cdot \right)$ is concave and by recalling the definition of β in (13). Note that $H_{b}\left(\beta \right)$ tends to zero when β tends to zero.

Since the decoder needs to recover *M* with high probability, Fano’s inequality implies the existence of some sequence $\left\{\epsilon_{n}\right\}$ tending to zero with the blocklength such that

(19) $$n(R-\epsilon_{n})\leq I(M;Y^{n}).$$

From these two inequalities, we obtain

(20)n(R−ϵn−Hb(β))≤I(M;Yn)−H(Sn|M,Yn)(21)=I(M;Yn,Sn)−I(M;Sn|Yn)−H(Sn|M,Yn)(22)=I(M;Yn,Sn)−H(Sn|Yn)(23)=I(M;Yn|Sn)−H(Sn|Yn)(24)=H(Yn|Sn)−H(Sn|Yn)−H(Yn|Sn,M)(25)=H(Yn)−I(Yn;Sn)−H(Sn)−I(Yn;Sn)−H(Yn|Sn,M)(26)=H(Yn)−H(Sn)−H(Yn|Sn,M).

Note that, above, we only used the chain rule and the fact that $M⊥\;⊥S^{n}$ (i.e., that *M* and $S^{n}$ are independent). The three terms in the last line can be bounded or simplified as follows:

(27)H(Yn)≤n(28)H(Sn)=H(S1)+(n−1)H({Si})(29)H(Yn|Sn,M)=H(Yn|Sn,M,Xn)(30)=H(Yn|Sn,Xn)(31)=∑i=1nH(Yi|Si,Xi)(32)=nπ0Hb(ϵ0)+π1Hb(ϵ1).

The converse now follows from (26), (27), (28), and (32).

### 4.2. Direct Part

For the direct part of the theorem, we assume that the state sequence is revealed to the encoder strictly causally and consider a Block-Markov coding scheme with backward decoding. Consider *b* blocks, each of *k* channel uses. Label the length-*k* typical state sequences $1,\ldots ,2^{k(H\left(\left\{S_{i}\right\}\right)+\epsilon )}$. For each block, randomly generate a codebook with $2^{k\tilde{R}}$ independent codewords (with $\tilde{R}$ to be specified later), each having *k* independent Bernoulli(1/2) components. In the first block, use the entire codebook to send a message of $k\tilde{R}$ bits. As to the transmission in Block *i* for i≥2, first check whether or not the state sequence in the previous block, namely, ${\left(s^{k}\right)}_{i-1}$, was typical. If it was, set $\ell_{i-1}$ to be the label that is assigned to it; else, set $\ell_{i-1}=1$. Use the generated codebook to send both $\ell_{i-1}$ and $m_{i}$, the latter consisting of $k\left(\tilde{R}-\left(H\left(\left\{S_{i}\right\}\right)+\epsilon \right)\right)$ information bits, so that the total number of bits matches the size of the codebook.

After completing the transmission in all *b* blocks, add one extra block to transmit $\ell_{b}$. To this end, the transmitter—ignoring any information about the states in this extra block—uses the Gilbert–Elliott channel to send $\ell_{b}$ (which we view as a message) and nothing else. The length of the extra block may be larger than *k*, but—as long as the Shannon capacity of our channel is positive—this will not affect the overall rate when we choose *b* to be very large. We will address this caveat shortly. However, first, we discuss the decoding.

To decode, we begin with the last bock to decode $\ell_{b}$ and thus recover ${\left(s^{k}\right)}_{b}$. This task will be accomplished successfully provided that the last extra block is sufficiently long. With the state information ${\left(s^{k}\right)}_{b}$ at hand, we then decode both $\ell_{b-1}$ and $m_{b}$ from Block *b*. Both can be decoded correctly with high probability (as *k* grows large) provided that

(33) $$\tilde{R}<I\left(X_{i};Y_{i}|S_{i}\right)=1-\left(\pi_{0}H_{b}\left(\epsilon_{0}\right)+\pi_{1}H_{b}\left(\epsilon_{1}\right)\right).$$

We continue this procedure backwards: By the time we get to decoding Block *i*, we will have already reliably recovered ${\left(s^{k}\right)}_{i}$ in Block (i+1), so we could use it as decoder-side information. The overall information rate—when *b* tends to infinity—approaches

(34) $$\tilde{R}-H\left(\left\{S_{i}\right\}\right)-\epsilon ,$$

which can indeed be made arbitrarily close to (14).

We have now established that, provided that $\tilde{R}$ is appropriately chosen, as *k* grows large, the probability of the decoder correctly decoding both the message and $s^{kb}$ tends to one. It only remains to note that this also means that the symbol error rate β is small. Indeed, having ${\hat{S}}^{kb}=S^{kb}$ with high probability guarantees that the symbol error rate in the first *b* blocks is close to zero, whereas the influence of the extra block (whose symbol error rate will typically be high, since we do not attempt to decode the states there) becomes negligible when we choose *b* to be large.

We now return to the caveat regarding the Shannon capacity and the extra block. We need to show that the Shannon capacity is positive whenever (14) is positive. (If (14) is not positive, there is no need for a direct part.) Recall that the Shannon capacity of the Gilbert–Elliott channel without any state information is $1-H(\left\{Z_{i}\right\})$, with $\left\{Z_{i}\right\}$ denoting the noise random variables, which form a Hidden Markov process. This Shannon capacity is positive unless $H(\left\{Z_{i}\right\})=1$ bit. Our concern regarding the caveat is thus only when $H(\left\{Z_{i}\right\})$ is 1.

In Appendix A, we show the following:

**Proposition** **1.** *If $H(\left\{Z_{i}\right\})=1$, then*

1. *$\left\{Z_{i}\right\}$ are IID Bernoulli(1/2), and*
2. *either $\left\{S_{i}\right\}$ are IID or $\epsilon_{0}=\epsilon_{1}=1/2$.*

If $\left\{Z_{i}\right\}$ are IID Bernoulli(1/2) and $\left\{S_{i}\right\}$ are IID, then (14) cannot be positive because, in this case, it equals

(35) $$H\left(Z_{i}\right)-H\left(Z_{i}|S_{i}\right)-H\left(S_{i}\right)=-H\left(S_{i}|Z_{i}\right).$$

If $\epsilon_{0}=\epsilon_{1}=1/2$, then (14) is also (trivially) not positive. Hence, indeed, the Shannon capacity is positive whenever (14) is positive.

## Data Availability

No new data were created or analyzed in this study. Data sharing is not applicable to this article.

## Abbreviations

The following abbreviations are used in this manuscript:

BSC	Binary Symmetric Channel
IID	Independent and Identically Distributed

## Appendix A. Proof of Proposition 1

In this appendix, we prove Proposition 1. We start with the first part, which we formally state as follows:

**Lemma** **A1.** *If $\left\{Z_{i}\right\}$ is a stationary process taking values in the binary set {0,1}, and if its entropy rate is* 1*, then it must be IID Bernoulli(1/2).*

**Proof.** For any positive integers ν and *ℓ*,

(A1)1ℓνHZ1ℓν=1ℓνHZ1ν,…,Z(ℓ−1)ν+1ℓν(A2)≤1ℓν∑i=1ℓHZ(i−1)ν+1iν(A3)=1ℓνℓHZ1ν(A4)=1νHZ1ν

where (A3) follows from the stationarity hypothesis. Letting *ℓ* tend to infinity so that the left-hand side of (A1) converges to the entropy rate, i.e., to 1, we obtain

(A5) $$1\leq \frac{1}{\nu }H\left(Z_{1}^{\nu }\right).$$

We obtain the reverse inequality by noting that $Z_{1}^{\nu }$ takes values in a set of cardinality $2^{\nu }$. We thus conclude that $Z_{1}^{\nu }$ is equiprobably distributed, and its components are thus IID Bernoulli(1/2). Since ν can be any positive integer, we conclude that $\left\{Z_{i}\right\}$ is an IID Bernoulli(1/2) process. □

We next show the remaining part of Proposition 1:

**Lemma** **A2.**
*If $\left\{Z_{i}\right\}$ are IID Bernoulli(1/2), and if $\epsilon_{0}$ and $\epsilon_{1}$ are not both equal to 1/2, then $\left\{S_{i}\right\}$ must be IID.*

**Proof.** We assume $\pi_{0},\pi_{1}\in \left(0,1\right)$; otherwise, $\left\{S_{i}\right\}$ are all deterministic and trivially IID. We also assume, without loss of generality, that

(A6) $$\epsilon_{0}<\frac{1}{2}.$$

For $Z_{i}$ to be Bernoulli(1/2), we must then have

(A7) $$\epsilon_{1}>\frac{1}{2}.$$

It then follows that the equation

(A8) $$\pi \epsilon_{0}+\left(1-\pi \right)\epsilon_{1}=\frac{1}{2}$$

has a unique solution in π, which must be $\pi_{0}$ as in the stationary distribution (2). Because $Z_{i}$ is Bernoulli(1/2) and $S_{i}$ is Bernoulli$\left(\pi_{1}\right)$, we can use Bayes’s rule to express the conditional distribution of $S_{i}$ given $Z_{i}=1$ as

(A9)PSi|Zi=1=2ϵ0π0,2ϵ1π1(A10)≠(π0,π1),

where the inequality follows from (A6) and (A7). Let M denote the (matrix form of the) Markov kernel $P_{S_{i+1}|S_{i}}$. We next assume that M is nonsingular and show that it leads to a contradiction. If M is nonsingular, then

(A11)PSi+1|Zi=1=2ϵ0π0,2ϵ1π1M(A12)≠(π0,π1)M(A13)=(π0,π1),

where the inequality follows from (A10) and the nonsingularity of M, and the last equality holds because $\left(\pi_{0},\pi_{1}\right)$ is the stationary distribution corresponding to M. Recalling that (A8) is only satisfied when π equals $\pi_{0}$, we conclude that

(A14) $$P_{Z_{i+1}|Z_{i}=1}\left(1\right)=P_{S_{i+1}|Z_{i}=1}\left(0\right)\;\epsilon_{0}+P_{S_{i+1}|Z_{i}=1}\left(1\right)\;\epsilon_{1}\neq \frac{1}{2},$$

which contradicts our assumption that $\left\{Z_{i}\right\}$ are IID Bernoulli(1/2). We thus conclude that the Markov kernel M must be singular, which, in the 2×2 case, means that its two rows are identical, and that $\left\{S_{i}\right\}$ are hence IID. □
